# EPIC: A Machine-Learning Framework for Product-Dependent Behavior in β‑Glucosidases

**DOI:** 10.1021/acsomega.6c04226

**Published:** 2026-07-02

**Authors:** Ali Malli, Denys Vasyutyn, Anuj Majumder, Jin Ryoun Kim

**Affiliations:** Department of Chemical and Biomolecular Engineering, 34242 New York University, 6 MetroTech Center, Brooklyn, New York 11201, United States

## Abstract

Enzyme activity is often modulated by interactions with small molecules. As molecules that can accumulate in the vicinity of enzymes, products can directly influence enzymatic activity and give rise to diverse enzyme response behaviors. Despite this biological importance, relatively few computational tools explicitly model enzyme–product interactions and characterizing such interactions typically relies on resource intensive experimental assays, thereby limiting enzyme mining and engineering efforts. Here, we present Enzyme–Product Interaction Classifier and Curve Predictor (EPIC), a machine learning framework that predicts glucose-dependent relative activity profiles of β-glucosidases (BGLs) from information on amino acid sequence and assay conditions. We curated a dataset of 105 unique BGL sequences for glucose response prediction. We formulate this problem as both a classification task, assigning enzymes to different product response classes, and a regression task, modeling full relative activity-glucose concentration profiles. Across extensive cross-validation, EPIC demonstrates more balanced classification performance across all glucose-response classes than a naïve sequence-identity-based baseline, attaining an *F*_1_-score of 0.577 ± 0.022 and an accuracy of 0.594 ± 0.024. EPIC substantially outperforms this baseline in predicting glucose concentration-dependent activity changes, capturing both monotonic and nonmonotonic response trends (Spearman’s ρ = 0.659 ± 0.025). Using ancestral and other enzymes beyond the original dataset, EPIC generalizes to unseen sequences and a range of response behaviors. Together, these results establish EPIC as a scalable framework for modeling relative enzyme activity in the context of enzyme–product interactions. Our study also demonstrates that classification and regression offer complementary perspectives, capturing the complex glucose-response behaviors of BGLs.

## Graphical abstract

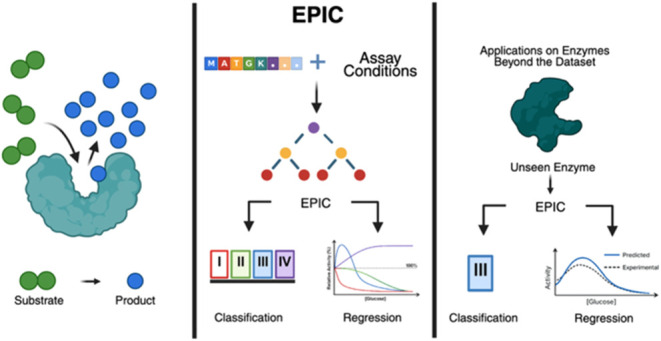

## 1 Introduction

Understanding protein-small molecule interactions is crucial in a wide range of biological processes including cellular metabolism, drug design, and enzyme engineering.^1,2^ Characterization of these interactions remains a challenge due to their inherent complexity, but there has been a growing interest in the development of computational and machine learning (ML) models that can predict consequences of these interactions.^1,3–5^ These efforts have primarily focused on enzyme–substrate reactions (*k*_cat_, *K*_m_, or *k*_cat_/*K*_m_).^5–9^ Computational or ML models have also been developed to predict enzyme inhibition by small molecules (*K*_i_)^7,10–12^ and receptor inhibition by drug-like molecules (IC_50_).^13–16^ However, less attention has been given to enzyme–product interactions which are manifested in many catalytic reactions as product inhibition and serve as a means of modulating enzyme activity in biological regulations. As molecules that can accumulate in the vicinity of enzymes, products can directly influence enzyme activity. Direct interactions between enzymes and products can substantially alter reaction kinetics, limit pathway flux, and constrain large-scale biochemical processes.^17^

Enzyme–product interactions play a prominent role in the enzymatic conversion of plant biomass into fuels and value-added chemicals.^18,19^ Lignocellulosic biomass represents an abundant and renewable feedstock, but its utilization usually relies on its depolymerization into fermentable sugars.^20^ In this context, cellulose is hydrolyzed by cellulolytic enzyme systems into glucose, which can then be metabolized by yeast, bacteria, and algae to produce bioethanol and other biofuels.^21,22^ The efficiency of this conversion process is strongly dependent on β-glucosidases (BGLs), a cellulase enzyme that catalyzes the hydrolysis of terminal nonreducing β-d-glucosyl bonds from cellobiose to release β-d-glucose.^23^ Despite their essential role, BGLs often represent a kinetic bottleneck in biomass degradation. The product of the enzymatic reaction, glucose, may inhibit BGL activity, reducing catalytic turnover and rendering cellobiose hydrolysis the rate-limiting step in biorefinery.^24^ While many characterized BGLs suffer from this issue, other BGL enzymes exhibit glucose tolerance or even glucose-stimulated activity.^25^ Recent modeling efforts aimed at predicting BGL performance in hydrolysis reactions have begun to emerge. For example, Erkanli and colleagues (2025) developed a temperature-dependent ML model to predict the catalytic efficiency of BGLs, providing insights into thermal effects on catalysis.^26^ However, no model has explicitly accounted for enzyme–product interactions, such as glucose inhibition, tolerance, or stimulation, which are important for accurately capturing enzyme behavior during catalysis.

When grouped by sequence and structural features, most characterized BGLs fall within glycoside hydrolase (GH) families 1 and 3, with other BGLs found in GH families 2, 5, 9, 30, 39, and 116.^27^ Notably, the GH1 BGLs are generally more tolerant to glucose than the GH3 BGLs, with some GH1 enzymes displaying glucose-stimulated activity.^28,29^ These broad trends are often attributed to shared catalytic mechanisms arising from conserved sequence and structural features within GH families. However, simple generalizations based on GH family membership are insufficient to reliably predict the glucose-response behavior of individual BGLs. Although GH3 enzymes are more frequently inhibited and GH1 enzymes are more often tolerant, these tendencies are weak and punctuated by notable exceptions.^29^ Another classification scheme to distinguish glucose-tolerant from glucose-inhibited BGLs uses a *K*_i_ threshold of 100 mM,^25^ but does not account for stimulatory behavior or the fact that the same BGL can shift its glucose-response behavior under different substrate concentrations.^30^

Extensive experimental and computational studies have sought to elucidate the molecular determinants underlying these diverse glucose response behaviors.^29,31–38^ Structural comparisons and mutational analyses of GH1 enzymes with distinct glucose-response profiles suggested that residues lining the entrance and interior of the substrate channel can form auxiliary glucose-binding sites that modulate inhibition, tolerance, and stimulation.^29^ According to this study, when glucose preferentially occupies the active site, inhibition dominates, whereas binding to distal sites along the channel can mitigate product inhibition or induce stimulation through transglycosylation.^29^ Docking and crystallographic studies of highly tolerant BGL variants also suggested that deep binding pockets external to the active site can sequester the interference of glucose with catalysis, with reductions in pocket size correlating with diminished tolerance.^33^ Complementary molecular dynamics-based analyses have proposed different mechanisms, including enhanced glucose expulsion via coordinated residue motions for tolerance (“slingshot” mechanisms),^38^ glucose-induced crowding of the active-site tunnel that obstructs water access,^39^ and conformational rearrangements that favor cellobiose binding over glucose binding at the catalytic site.^36^ Collectively, these studies underscore that glucose response mechanisms are highly complex and, in some cases, yield conflicting mechanistic interpretations across different BGLs even within the same GH family. As a result, accurate characterization of glucose interactions with BGL typically requires direct experimental measurements using purified enzymes. Such assays are labor-intensive, time-consuming, and costly, limiting their scalability and slowing enzyme discovery and engineering efforts.

Taken together, the absence of a predictive model for enzyme–product interactions, the limitations of existing threshold-based classifiers, and the lack of a unified mechanistic framework motivate a data-driven approach. To address these challenges, we present Enzyme–Product Interaction Classifier and Curve Predictor (EPIC), an ML framework designed to predict enzyme behavior due to product interactions. EPIC focuses specifically on predicting the glucose-dependent behavior in BGLs, directly from sequence information and substrate concentrations as primary features. We did not intend to develop a predictor for enzyme–product interactions across a broad range of functionally unrelated enzymes (referred to as a global predictor). This is because the relevant data are not well available in public databases like BRENDA.^40^ More importantly, recent studies have shown that global ML models trained across diverse enzyme families often underperform when applied to family specific datasets as they do not sufficiently cover local regions of the sequence space.^5,26,41^

The novelty of EPIC lies in the formulation of enzyme–product interactions as a predictive task linking sequence representations and assay conditions to full product-dependent relative activity profiles rather than in the development of new ML architectures. Accordingly, EPIC aims to provide a scalable and data-driven alternative to exhaustive experimental screening, enabling rapid identification of glucose-tolerant and glucose-stimulated BGLs for bioindustrial applications, as needed. While initially framed as a classification task, we demonstrated that EPIC can be operated in regression mode to gain a more comprehensive prediction regarding the relative activity profile with varying glucose concentrations.

## 2 Methods

### 2.1 Collection and Curation of the β-Glucosidase Dataset

A manual PubMed search was conducted in November 2025 to collect information on the classification of BGLs based on their responses to glucose, the product of their hydrolysis reactions. Amino acid sequences for the collected BGLs were retrieved from UniProt,^42^ EMBL,^43^ or other databases^44,45^ as needed. Both wild-type enzymes and their mutants, whether naturally occurring or engineered, were included. Each BGL was classified into one of four classes based on the effect of glucose on their enzymatic activity toward the chromogenic substrate p-nitrophenyl-β-d-glucopyranoside (pNPG), following the classification scheme described by Salgado and colleagues (2018). Accordingly, BGLs can be inhibited, tolerant, or stimulated by glucose. Stimulation can be manifested by two behaviors: increasing activity at low glucose concentrations followed by a decrease at high glucose concentrations or a monotonous increase in activity even at high glucose concentrations.^25^ This dataset is hereafter referred to as the classification dataset (Table S1).

Besides the classification data above, quantitative data describing the relative activity of BGLs as a function of glucose concentration were extracted from glucose-response plots in the published papers using WebPlotDigitizer (version 5.2; https://automeris.io/WebPlotDigitizer). Activity values for each enzyme were normalized to the activity measured at 0 mM glucose. The substrate concentration used in the glucose response assay was also recorded. These data entries comprised the regression dataset (Table S2). For each enzyme entry in Tables S1 and S2, there is one substrate concentration.

The classification dataset contains 105 unique BGL sequences, including 63 wild-types and 42 mutants. The average sequence length is 491 ± 102 amino acids, and the mean pairwise sequence identity is 44 ± 12%. The regression dataset comprises 919 data entries of relative activity measured across various glucose concentrations for the same set of BGL sequences.

### 2.2 Sequence Encodings and Feature Vectors

Both proposed prediction tasks start with generating sequence encodings for enzymes. We first utilized protein language models (pLMs) pretrained on a large array of protein sequences. Two pLMs were considered in inference mode with frozen parameters: ESM-2 (650 M parameters)^46^ and ProstT5.^47^ These models were selected because they capture evolutionary and functional information directly from large-scale protein sequence corpora and have demonstrated strong performance across diverse protein property prediction tasks.^5,48,49^ While ESM-2 is trained on amino acid sequences and primarily encodes sequence-derived evolutionary patterns, ProstT5 is a bilingual model trained to understand and translate between both protein sequences and 3D structures. The inclusion of both embeddings therefore allows for the comprehensive evaluation of complementary representations spanning sequence-only and sequence-structure-informed feature spaces. Briefly, each amino acid was converted into a multidimensional vector from the final encoder layer, and the resulting vectors were averaged using mean pooling. The final enzyme representations were 1280-dimensional and 1024-dimensional vectors using ESM-2 and ProstT5 respectively. Computations were performed on New York University’s High Performance Computing Greene cluster using NVIDIA V100 GPUs (16 GB memory, CUDA v11.1.74) when available; otherwise, analyses were executed on CPU (1 core).

In addition, enzyme sequences were encoded into overlapping *n*-grams (*n* = 3) of amino acids using a character-level vectorization approach. The Trigrams frequencies were normalized by the total number of all possible Trigrams per sequences to generate relative frequency vectors. The final Trigrams representation of each sequence was an 8000-dimensional vector.

For the classification model, these embeddings were appended with the substrate (pNPG) concentration for each entry and used as features. As for the regression model, they were concatenated with the glucose concentration and the substrate concentration for each entry.

### 2.3 Model Settings

#### 2.3.1 Classification Model

The classification dataset was split into 5-folds using stratified partitioning to preserve class proportions. To ensure that the reported performance is not limited to the original data split, we repeated the 5-fold split 20 times using different random seeds. Within each repeat, each sample appeared exactly once in the test set across the 5 folds. We tested four machine learning models using *sklearn* v1.7.2: random forest (RF), ExtraTrees, gradient boosting classifier (GBC), and support vector machine (SVM). RF, ExtraTrees, and SVM accounted for class imbalance via their class weight argument.

#### 2.3.2 Regression Model

The regression dataset was split into grouped 5-folds by enzyme sequence ensuring that the data points from the same sequences are never partitioned across training and test sets. Enzyme identifiers were used as grouping variables, and folds were constructed over unique enzyme sequences rather than individual data points. The training was repeated five times to ensure that each fold acted as a test set once. Like the classification model, we repeated the 5-fold cross-validation 20 times using different random seeds, resulting in 100 train-test evaluations per model configuration. We tested four machine learning models using *sklearn* v1.7.2: RF, ExtraTrees, gradient boosting regressor (GBR), and support vector regressor (SVR). Tree-based models were initially trained using default hyperparameters and random seeds for reproducibility. Finally, to enforce physically meaningful predictions, predicted relative activity values corresponding to zero glucose concentration were explicitly anchored to 1 prior to performance evaluation.

### 2.4 Calculation of Evaluation Metrics

#### 2.4.1 Classification Model

Performance was assessed using macro-averaged *F*_1_-score computed with the test sets per repeat with *C* = 4 classes, as in eqs 1–3. TP, FP, and FN correspond to the numbers of true positives, false positives, and false negatives, respectively.

1 $${F_{1}}_{\mathrm{macro}}=\frac{1}{C}\sum_{c=1}^{C}\frac{2\cdot {\mathrm{Precision}}_{c}\cdot {\mathrm{Recall}}_{c}}{{\mathrm{Precision}}_{c}+{\mathrm{Recall}}_{c}}$$

2 $${\mathrm{Precision}}_{c}=\frac{{\mathrm{TP}}_{c}}{{\mathrm{TP}}_{c}+{\mathrm{FP}}_{c}}$$

3 $${\mathrm{Recall}}_{c}=\frac{{\mathrm{TP}}_{c}}{{\mathrm{TP}}_{c}+{\mathrm{FN}}_{c}}$$

Moreover, we considered accuracy, defined as the fraction of correctly classified sequences across all *n* sequences in the test sets (eq 4). I(·) is an indicator function that returns 1 if the prediction (*ŷ*_*i*_) is consistent with the actual class (*y*_*i*_) and 0 otherwise.

4 $$\mathrm{Accuracy}=\frac{1}{n}\sum_{i=1}^{n}I\left(y_{i}={ŷ}_{i}\right)$$

For each repeat *r*, all predictions from the 5 cross-validation folds were pooled such that each sequence contributed exactly one prediction. Then, a confusion matrix $M^{\left(r\right)}\in N^{C\times C}$ was computed from {(*y*_*i*_, *ŷ*_*i*_^(*r*)^)}_*i*=1_^*n*^ where each entry *M*_*ab*_^(*r*)^ counts sequences of true class *a* predicted as class *b* across *n* unique BGL sequences. By construction, Σ_*a*,*b*_ *M*_*ab*_^(*r*)^ = *n* for every repeat. Reported summary metrics are presented as an average (Ω̅) with their standard deviation (σ_Ω_) across *N* repeats (eqs 5 and 6). Ω^(*r*)^ denotes either *F*1_macro_ or accuracy for repeat *r*.

5 $$\mathrm{\Omega ̅}=\frac{1}{N}\sum_{r=1}^{N}\Omega^{\left(r\right)}$$

6 $$\sigma_{\Omega }=\sqrt{\frac{1}{N-1}\sum_{r=1}^{N}{\left(\Omega^{\left(r\right)}-\mathrm{\Omega ̅}\right)}^{2}}$$

#### 2.4.2 Regression Model

Model performance was quantified using complementary metrics aiming to capture both the shape and magnitude of predicted glucose inhibition/stimulation/tolerance profiles. All metrics were computed on a per-enzyme basis using reported (*y*_*i*_) and predicted (*ŷ*_*i*_) relative activity values measured across *n* glucose concentrations, denoted as {(*y*_*i*_, *ŷ*_*i*_)}_*i*=1_^*n*^. Our selected metrics included:

- Spearman’s rank correlation coefficient (ρ) in eq 7. It was used to quantify the monotonic agreement between the predicted and reported enzyme response curves, independent of absolute scale. In other words, ρ captures whether the predicted activity profile correctly reproduces the ordering of activity values across increasing glucose concentrations.
7 ρ=corr(rank(y),rank(ŷ))
  where ρ ∈ [ −1,1] with ρ = 1 indicating perfect monotonic agreement and ρ = −1 indicating perfectly reversed monotonic ordering.
- Root mean square error (RMSE) in eq 8. It was calculated between predicted and reported relative activity to assess deviations in magnitude and reflect the overall accuracy of predicted enzyme response profiles.
8 $$\mathrm{RMSE}=\sqrt{\frac{1}{n}\sum_{i=1}^{n}{\left(y_{i}-{ŷ}_{i}\right)}^{2}}$$
- Affine-transformed RMSE (RMSE_aff_) in eq 9. We introduced this metric to compare the shapes of predicted and reported curves, by decoupling absolute scaling and offset differences. Accordingly, predicted activity values were linearly rescaled via least-squares fitting prior to RMSE calculation as in eq 10, allowing for the independent evaluation of shape agreement with the reported values. *a* and *b* are fitting parameters that correct for scale and offset, respectively.
9 $${\mathrm{RMSE}}_{\mathrm{aff}}=\sqrt{\frac{1}{n}\sum_{i=1}^{n}{(y_{i}-\left(a\cdot {ŷ}_{i}+b\right))}^{2}}$$

10 $$\left(a,b\right)=\arg\min_{a,b}\sum_{i=1}^{n}{(y_{i}-\left(a\cdot {ŷ}_{i}+b\right))}^{2}$$

The final reported metrics (Ω̅) are the average of fold-level metrics within each cross-validation fold *k* over *K* folds and *N* repeats (eq 11).

11 $$\mathrm{\Omega ̅}=\frac{1}{N}\sum_{r=1}^{N}\left(\frac{1}{K}\sum_{k=1}^{K}\Omega_{r,k}\right)$$

### 2.5 Sequence-Identity-Based Baselines

To contextualize the performance of EPIC and assess whether its predictions exceed a naïve benchmark based on simple homology-based heuristics,^50^ we implemented a sequence-identity-based baseline for both regression and classification tasks. Pairwise sequence identity was computed between all enzyme sequences using global sequence alignment. Briefly, for each query enzyme in the test set, alignments were performed against all enzymes in the training dataset, and percent sequence identity was calculated as the fraction of identical residues over the aligned length. Then, the top 3 nearest neighbors in sequence identity space were identified. These nearest neighbors served as the reference set for baseline prediction.

For the classification task, the sequence-identity baseline assigns each query enzyme to the majority class among its top 3 nearest neighbors in the sequence identity space. In cases of ties, the class associated with the highest-identity neighbor was selected. This approach mirrors common homology-based annotation transfer strategies and provides a naïve benchmark for evaluating learned classifiers.^50,51^

For the regression task, the sequence-identity baseline predicts enzyme relative activity by transferring experimental measurements from the most similar training enzymes. Specifically, for a given query enzyme and glucose concentrations, predicted relative activity was computed as the average of the corresponding activities from the top 3 nearest neighbors. If a given glucose concentration was missing for a neighboring enzyme, its relative activity value was obtained using linear interpolation when the concentration fell within the experimentally measured glucose range. When the request concentration lay outside this range, the relative activity was assigned the value measured at the nearest boundary concentration.

### 2.6 Hyperparameter Optimization

For the classification (ProstT5 + SVM) and regression (Trigrams + GBR and ProstT5 + ExtraTrees) configurations selected for downstream analysis (e.g., generalization to unseen sequences, SHAP analysis, etc.), we performed targeted hyperparameter optimization to further enhance model performance and compare it with the sequence-identity-based baseline. For each model, hyperparameters were tuned using random search over model-specific parameter ranges. In each tuning run, 40 candidate hyperparameter combinations were sampled and evaluated using 5-fold cross-validation. For each candidate combination, the model was trained on four folds and evaluated on the remaining fold, and this procedure was repeated across all 5 folds. The average performance across the five folds was used to score each candidate combination. Macro-averaged *F*_1_-score was used to evaluate classification models and RMSE was used to evaluate regression models in order to prioritize accurate prediction of relative activity values across glucose concentrations. Rank-based and affine-invariant metrics were not used for optimization because they are insensitive to systematic scaling errors.

The procedure was repeated 20 times using different shuffled enzyme-level fold assignments. The 20 repeated 5-fold splits were used to quantify sensitivity to fold assignment. In each repeat, every sample appeared once in the test set, but the composition of each fold varied across repeats. Therefore, a given enzyme could be evaluated while different other enzymes were excluded from the training set, allowing us to estimate how dependent performance was on a particular partitioning of the small and imbalanced dataset. Each repeat therefore produced one best-performing hyperparameter combination based on its mean cross-validation score. Across the 20 repeats, the hyperparameter combination achieving the highest cross-validation score was selected as the final configuration. The selected configuration was then used in subsequent experiments such as benchmarking against the sequence-identity-baseline and generalizing to unseen sequences. This procedure avoids information leakage from evaluation folds during parameter tuning while reducing sensitivity to any single favorable data split. Finally, the optimal hyperparameters were used to retrain the model on the full dataset prior depositing it to the online repository.

### 2.7 Dimensionality Reduction

Protein language model embeddings are high-dimensional representations which can increase the risk of overfitting and compromise model stability when training supervised models on relatively small datasets. To assess whether our models experience these issues and, if so, to mitigate this effect, we applied principal component analysis (PCA) as an optional dimensionality reduction step prior to model training. PCA has previously been shown to preserve the dominant functional signal in pLM embeddings while improving robustness in low-data prediction tasks.^52^ When enabled, the enzyme representations were reduced to 50-dimensional vectors fitted using the training data and applied to the corresponding test data within each cross-validation fold. PCA was not applied to substrate and glucose concentrations.

### 2.8 Benchmarking Protocol

Select combinations of embedding types (ProstT5, ESM-2, Trigrams), ML regressors (RF, ExtraTrees, GBR, SVR), and PCA usage (enabled or disabled) were systematically evaluated. Performance summaries and per-repeat metrics were recorded for each combination to enable comparison across modeling choices.

### 2.9 Statistical Analysis and Hypothesis Testing

All statistical analyses were performed in Python using standard scientific computing libraries (NumPy and SciPy). Unless otherwise stated, reported metrics represent mean ± standard deviation across repeated cross-validation runs. To assess whether differences in model performance were statistically significant, we employed Wilcoxon signed-rank tests used to compare paired performance metrics (e.g., RMSE, ρ, etc.) between learned models and sequence-identity baseline methods across cross-validation repeats. All statistical tests were two-sided. Exact *p*-values are reported in text and figures. Statistical significance was assessed at a nominal threshold of *p*-value <0.05.

### 2.10 Cloning, Expression, and Enzyme Activity Measurement of BGL Ancestral Sequences

To test our model’s generalization to unseen sequences beyond our original dataset, we leveraged two novel BGL sequences previously developed by our group using ancestral sequence reconstruction (ASR). The ancestral genes were synthesized and cloned into a pET-30a­(+) vector by GenScript (Piscataway Township, NJ). The ASR and protein expression protocols were conducted as reported in our previous study.^26^

The glucose response activity curves of the ancestral sequences were determined by measuring the rate of hydrolysis of pNPG (Sigma-Aldrich, St. Louis, MO) in the presence of various glucose concentrations (0 mM, 50 mM, 250 mM, and 500 mM) at 70 °C. At this assay temperature, the BGL ancestral sequences were fully active due to their high thermostability. The reaction was conducted in citrate phosphate buffer (pH 6) with 2 mM pNPG as a substrate. Initial rates of hydrolysis were determined by measuring the release of p-nitrophenol (pNP) at 400 nm over time using a Varian Cary 50 UV–vis spectrophotometer (Palo Alto, CA) coupled with a Quantum Northwest temperature controller (Liberty Lake, WA). The reaction volume was 2.5 mL and the enzyme concentration in solution was 0.02 μM. The change in the molar extinction coefficient upon full hydrolysis of pNPG (Δε) was measured to be 1.812 mM^–1^cm^–1^. The relative activities were defined as the activity values at different glucose concentrations relative to the activity of the enzyme in the absence of glucose.

## 3 Results and Discussion

In the following sections, we evaluate EPIC across two prediction tasks: classification of enzymes into glucose-response classes and regression of full glucose-dependent relative activity curves (Figure 1). EPIC integrates sequence-derived representations from pLMs to support both tasks within a unified framework. The substrate concentration was included as an additional feature, as experimental studies showed that a change in substrate concentrations causes a shift in glucose-response behaviors across multiple BGLs (Figure S1).^26,30^ Substrate concentration might determine the competitive balance between substrate and glucose binding within and around the active site, thereby leading to distinct glucose responses. Other assay conditions like temperature and pH were not considered as the assays were usually conducted at their optimal values or because they were not consistently reported across the underlying studies. We first assess classification performance under different features and models. We then examine regression accuracy in predicting relative activity magnitudes and response trends, including comparisons against sequence-identity-based baselines and analyses with external test sets not included in the original dataset. The baselines reflect the homology-based assumption that enzymes with high sequence identity tend to share functional behavior.^53^ The baselines serve as naïve benchmarks that are highly relevant in this study, as our dataset contains closely related sequences due to shared BGL activity and point mutations. A similar baseline was previously used to benchmark ML models for predicting other enzyme activity parameters.^8,50^

**Figure 1:**
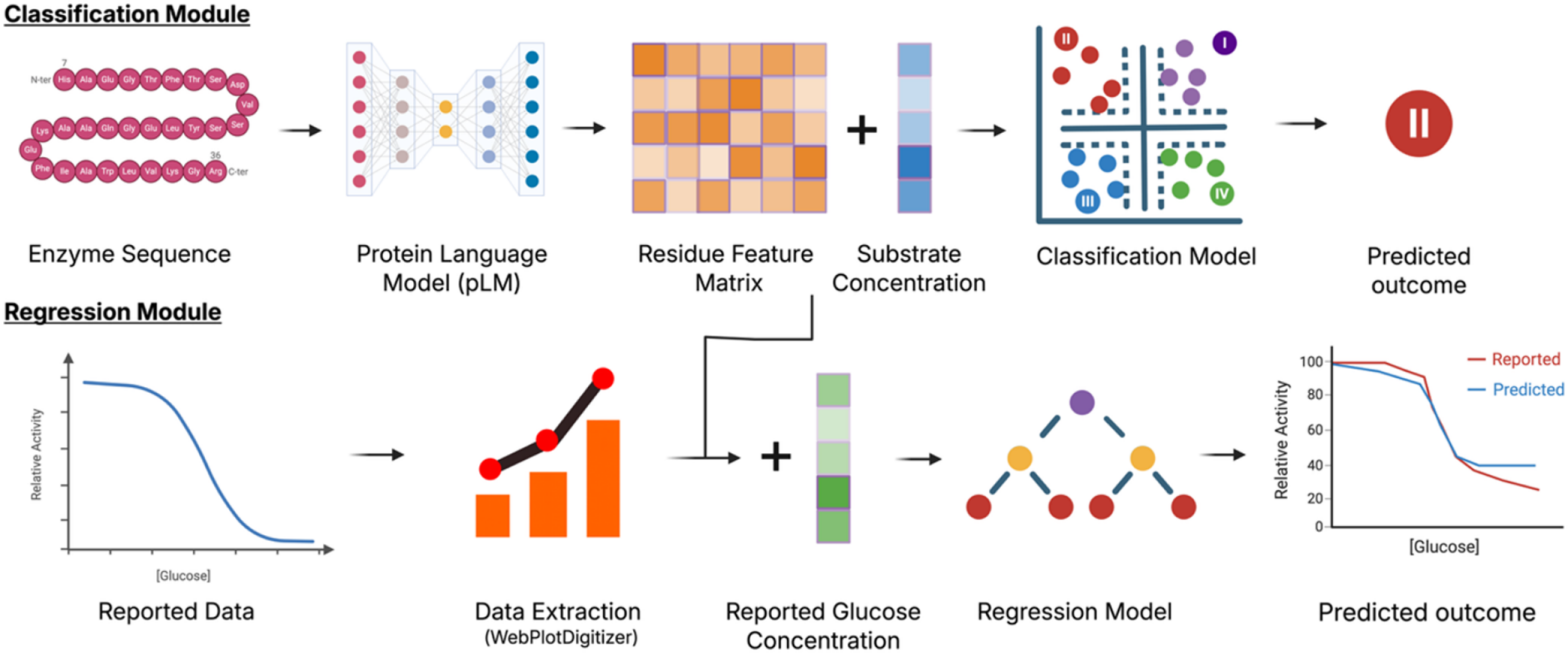
Overview of the EPIC framework for enzyme–product interaction modeling. EPIC integrates enzyme sequence information and assay context to support both classification and regression tasks. Enzyme sequences are encoded using a protein language model (pLM) and combined with substrate concentration features to predict glucose-response classes (top). Reported glucose-dependent activity data are extracted and paired with the classification feature matrix to train regression models that predict full relative activity curves (bottom).

### 3.1 Formulating EPIC as a Classification Task

We first evaluated whether glucose response of BGLs could be classified by EPIC from sequence and assay conditions. As shown in Table 1, each enzyme was assigned to one of four discrete glucose-response classes defined by Salgado and colleagues (2018): (I) BGLs strongly inhibited by low concentrations of glucose, (II) BGLs tolerant to low glucose concentrations, (III) BGLs stimulated by low glucose concentrations, followed by a decrease in activity at high glucose concentrations, and (IV) BGLs stimulated by both low and high glucose concentrations.^25^ To this end, we benchmarked a set of classical ML classifiers with default hyperparameters using three sequence encoding methods: pretrained pLMs (ProstT5 ^47^ and ESM-2 ^46^) and normalized amino-acid Trigrams frequencies. The substrate concentration was also included here as an input feature, as it varied substantially between studies across our database.

**Table 1:** Class Distribution in the EPIC Classification Dataset

class	number of entries
I	24
II	30
III	46
IV	5
Total	105

Across all encoding and model combinations, classification showed a reasonable performance with macro-averaged *F*_1_-scores and accuracies ranging between 0.45 and 0.56 and 0.54–0.6 respectively (Table 2). The best performing model, ProstT5 combined with an SVM classifier, achieved a macro *F*_1_-score of 0.558 ± 0.023 and an overall accuracy of 0.571 ± 0.025, and was subjected to hyperparameter optimization.

**Table 2:** Classification Performance^a^

		*F*_1macro_		accuracy	
encoding	model	mean	standard deviation	mean	standard deviation
ProstT5	RF	0.487	0.040	0.597	0.023
	ExtraTrees	0.473	0.041	0.594	0.022
	GBC	0.475	0.039	0.567	0.034
	SVM	**0.558**	**0.023**	**0.571**	**0.025**
ESM-2	RF	0.500	0.044	0.552	0.020
	ExtraTrees	**0.520**	**0.020**	**0.552**	**0.017**
	GBC	0.461	0.052	0.537	0.027
	SVM	0.515	0.022	0.516	0.022
Trigrams	RF	0.452	0.035	0.556	0.025
	ExtraTrees	0.456	0.048	0.562	0.030
	GBC	0.442	0.041	0.545	0.027
	SVM	**0.560**	**0.021**	**0.563**	**0.026**

a Mean and standard deviation of classification metrics across 20 rounds of five-fold cross-validation are reported. The best performing model for each encoding method is highlighted in bold.

After optimizing the hyperparameters of the ProstT5 + SVM classifier, direct comparison of the model was done against a baseline that predicts the class of each enzyme in the test set by majority voting among the three enzymes in the training set that share the highest pairwise sequence identity with the test enzyme. The model achieved a significantly higher macro *F*_1_-score (*p*-value = 2.67 × 10^–5^) than the baseline while the baseline retained a modest advantage in overall accuracy, albeit with no statistical significance (*p*-value = 5.27 × 10^–2^) (Figure 2A,B). With Class III being the largest class (Table 1, *n* = 46), overall accuracy is more sensitive to majority-class performance whereas macro *F*_1_-score penalizes class imbalance by weighting classes equally. Hence, these results indicate that while nearest-neighbor sequence identity remains a strong predictor of dominant class labels in Class III, the classification model incorporating both sequence and substrate concentration captures a more balanced performance across all glucose-response classes (Figure S2A,B). In other words, EPIC’s role should be interpreted as predicting enzyme behavior under specified assay conditions rather than predicting a context-independent enzyme property, although how well EPIC learns this context dependency relies on the composition of the current dataset.

**Figure 2:**
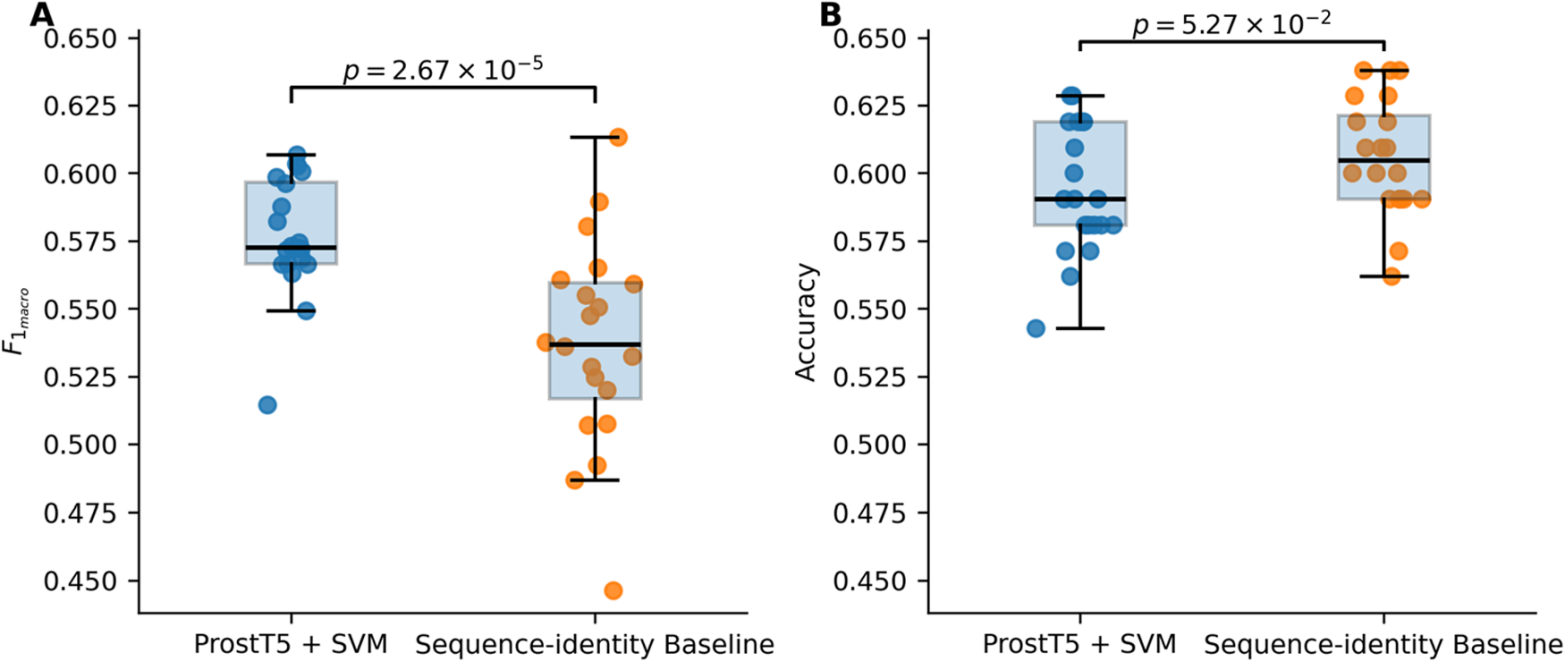
Distributions of (A) macro-average *F*_1_-score and (B) overall accuracy across 20 repeats of 5-fold cross-validation. Panels A and B compare the ProstT5-based SVM classifier against the sequence-identity baseline. *p*-values are from a paired two-sided Wilcoxon signed-rank test across repeats.

Due to the small size of Class IV, we assessed whether a simplified three-class formulation would provide a more robust alternative: Classes I and II were still treated as inhibited and tolerant, respectively, while Classes III and IV were grouped as stimulated. The optimal classifier for this task, ProstT5 + RF, performed comparably to the sequence-identity baseline, with no statistically significant difference in macro-F1 score or accuracy (Figure S3). These results indicate that local sequence identity may be sufficient to distinguish among the three classes. However, ML training using separate Class III and Class IV datasets may extract additional predictive features (especially those related to Class IV) that are not captured by sequence identity alone.

To understand the non-negligible performance of the baseline, we visualized the enzyme dataset in a two-dimensional sequence-identity space using multidimensional scaling (MDS) based on pairwise sequence identities (Figure S4). While enzymes from different glucose-response classes do not form well-separated clusters, they frequently occupy overlapping regions of the sequence space. Nevertheless, this overlap is structured in a way that enzymes with high sequence identity often share the same response class, particularly many Class I and III enzymes that occupy local regions of the space. This organization explains the non-negligible performance of the nearest-neighbor baseline.

Importantly, the four glucose-response classification represents a discretization of more continuous glucose response profiles. As such, enzymes with similar activity curves, but slightly shifted slopes may be assigned to different classes (e.g., tolerant instead of inhibited), despite minimal functional differences. Hence, this discretization introduces ambiguity at class boundaries that cannot be resolved from sequence information and substrate concentration alone.

### 3.2 Formulating EPIC as a Regression Task

The aforementioned observations motivated the shift toward a regression model that predicts the full glucose response curve. To move beyond mere class assignment and explicitly model the shape and magnitude of glucose response curves of BGLs, we formulated EPIC as a regression task and evaluated a broad sweep of sequence encodings and regression models. Specifically, we benchmarked the previous encodings (ProstT5, ESM-2, and Trigrams) paired with four regression algorithms (RF, ExtraTrees, GBR, and SVR) using 5-fold cross-validation repeated 20 times. Similar to the classification module, substrate concentration was retained in the feature vector for all further experiments. Figure 3 summarizes regression performance across all encoding-model combinations.

**Figure 3:**
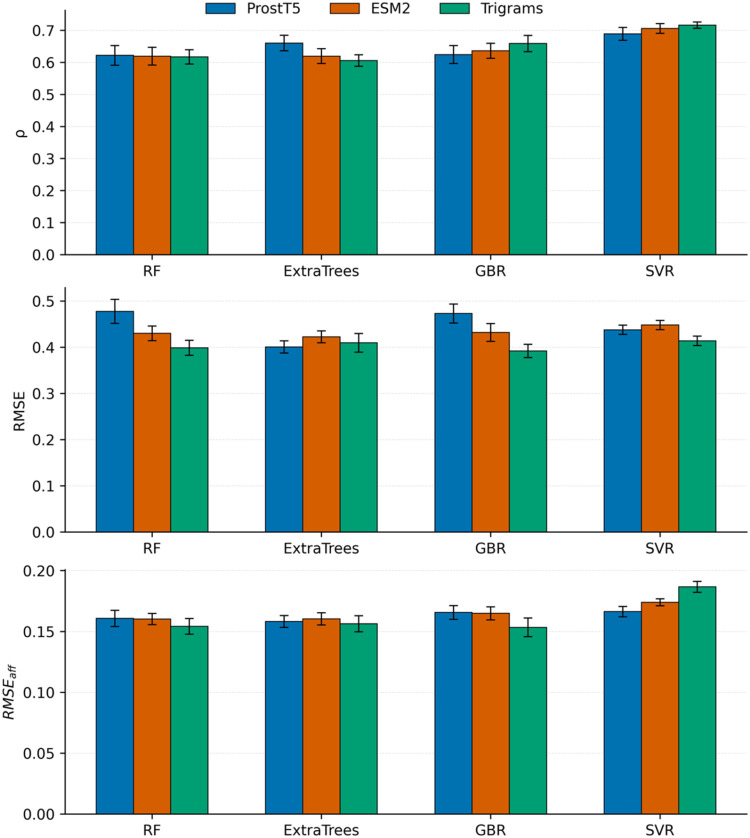
Regression performance across model and embedding combinations. Mean ± standard deviation of ρ (top), RMSE (middle), and RMSE_aff_ (bottom) over 20 repeats of 5-fold cross-validation are shown.

Overall, SVR consistently achieved the highest Spearman’s rank correlations with peak performance approaching ρ ≈ 0.7. This indicates that SVR most reliably preserves the monotonic ordering of relative activity across glucose concentrations, suggesting accurate captures of inhibitory or stimulatory trends independent of absolute scale. In contrast, tree-based methods generally exhibited slightly lower rank correlations but competitive error metrics. In terms of enzyme representations, ProstT5 showed the highest ρ with ExtraTrees while Trigrams outperformed when coupled with GBR and SVR.

RMSE values revealed a different pattern. Trigram-based models achieved the lowest absolute errors, except when using ExtraTrees, indicating improved prediction of relative activity magnitudes. While raw RMSE values correspond to 0.4–0.5 (i.e., 40–50% relative error in predicted relative activity magnitude), this error range is expected given the limited size of the training dataset. We therefore additionally calculated an affine-transformed RMSE to decouple curve shape from linear scaling effects. Across all encoding and model combinations, affine-transformed RMSE values were comparatively small, suggesting that our approaches broadly capture response shapes even when absolute magnitudes differ.

Considering all three metrics collectively, we selected the Trigrams + GBR model for downstream analysis. Although, when paired with Trigrams, SVR achieved the highest rank correlations, the advantage of this combination was marginal relative to those with tree-based regressors and came at the cost of higher affine-transformed RMSE. Meanwhile, the Trigrams + GBR model achieved a favorable balance across all three metrics, combining the lowest affine-transformed RMSE (RMSE_aff_ = 0.153 ± 0.008), the lowest absolute error (RMSE = 0.392 ± 0.014), and a strong monotonic agreement (ρ = 0.659 ± 0.025). Importantly, this configuration enables direct attribution of sequence features for biological interpretation through feature-importance and SHapley Additive exPlanations (SHAP) analysis of the Trigrams. ProstT5 + ExtraTrees was retained as a complementary embedding-based model due to its stable performance across metrics.

Following hyperparameter optimization, both regression models were benchmarked against a sequence-identity-based baseline using ρ, RMSE, and RMSE_aff_ as metrics (Figure 4). For each test enzyme, we identified the top three most similar enzymes in the training set based on sequence identity and reconstructed the test enzyme’s glucose response curve by spline-interpolating and averaging the neighbors’ curves at the test glucose concentrations. This approach provides a stringent reference that exploits local homology without fitting any parametric model.

**Figure 4:**
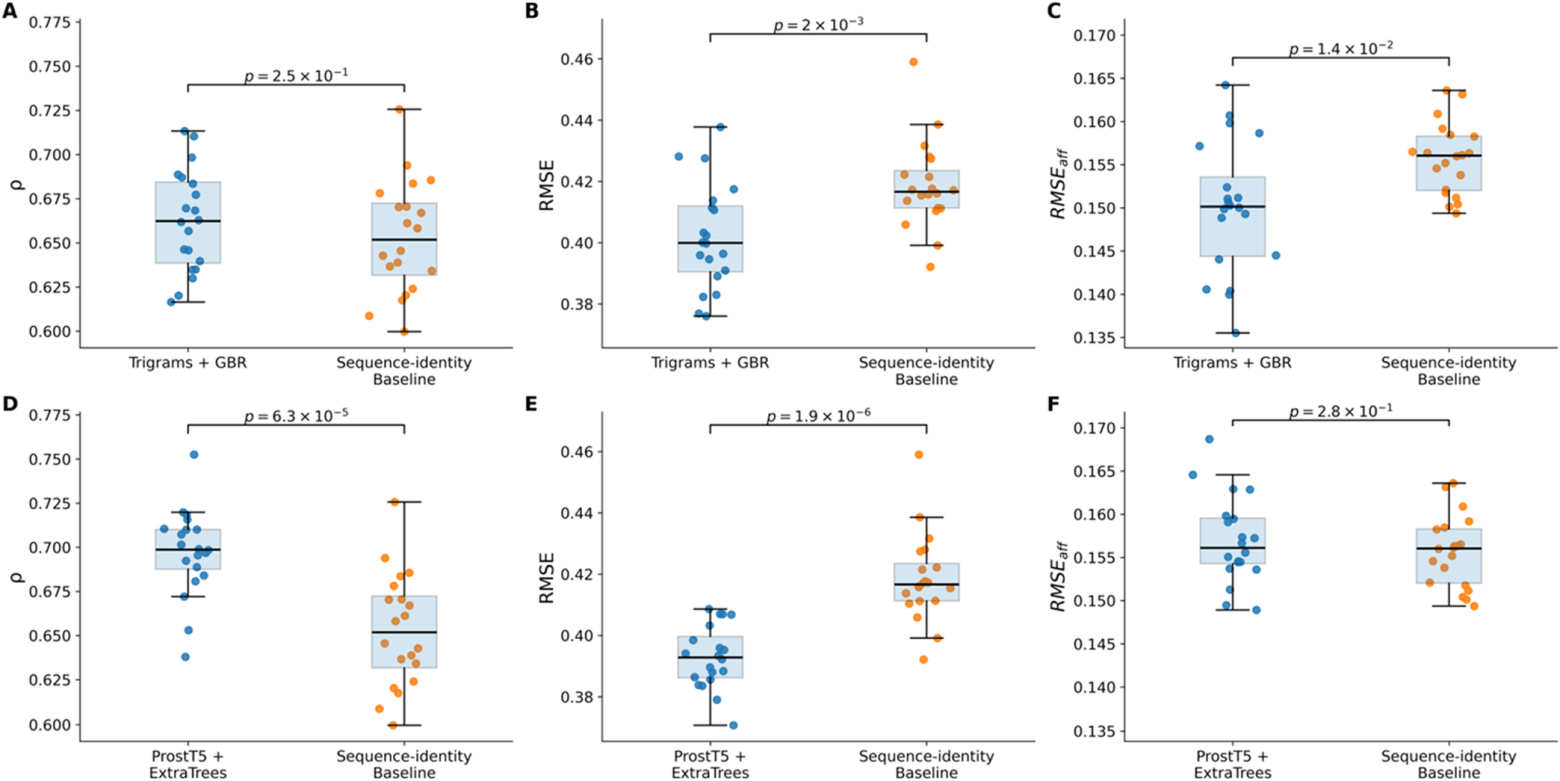
Distribution of ρ, RMSE, and RMSE_aff_ across 20 repeats of 5-fold cross-validation for the (A–C) Trigrams + GBR and (D–F) ProstT5 + ExtraTrees regressors against the sequence-identity baseline. *p*-values are from a paired Wilcoxon signed-rank test across repeats.

For Trigrams + GBR, the model showed a significantly reduced affine-transformed RMSE relative to the baseline (*p*-value = 1.4 × 10^–2^; Figure 4C) while the ProstT5 + ExtraTrees did not (*p*-value = 2.8 × 10^–1^; Figure 4F). Meanwhile, Trigrams + GBR and ProstT5 + ExtraTrees achieved significantly lower RMSE than the baseline (Trigrams + GBR: *p*-value = 2 × 10^–3^, Figure 4B; ProstT5 + ExtraTrees: *p*-value = 1.9 × 10^–6^, Figure 4E), indicating substantially improved prediction of relative activity magnitudes across glucose concentrations. These gains reflect the ability of regression models to integrate magnitude information across the full training set rather than relying on a small number of nearest neighbors. As for Spearman’s ρ, Trigrams + GBR did not differ significantly from the baseline (*p*-value = 2.5 × 10^–1^; Figure 4A) while ProstT5 + ExtraTrees exhibited a statistically significant increase in rank correlation (*p*-value = 6.3 × 10^–5^, Figure 4D). Accordingly, local sequence identity is informative of glucose response trends, with regression models offering additional gains depending on the sequence representations and choice of regressors.

Together, these results demonstrate that, although the baseline captures overall trends to some extent, regression models provide consistently more accurate predictions of relative activity magnitudes. Particularly, ProstT5 + ExtraTrees appears effective at correcting biases introduced by naïve interpolation, especially when neighboring enzymes differ in scale and rank correlations despite similar response shapes.

We also examined representative regression predictions spanning good, intermediate, and poor performance cases of the Trigrams + GBR and ProstT5 + ExtraTrees regressors (Figure S5). For well-predicted enzymes, the model accurately captured both the overall magnitude and the trend of relative activity across glucose concentrations (Figure S5A,D). Intermediate cases generally preserved the correct response shape but exhibited modest deviations in scale (Figure S5B,E) while poorly predicted examples showed larger discrepancies in activity as well as altered slopes or curvatures (Figure S5C,F). These examples highlight the strengths and limitations of the regression models and contextualize the RMSE- and correlation-based performance metrics reported above.

### 3.3 Dimensionality Reduction

To assess the potential risk in EPIC predictions arising from the high feature-to-sample ratio and overfitting in high-dimensional feature spaces, we applied PCA to the input feature representations for both the regression and classification modules of EPIC.^52^ For the regression task using the Trigrams + GBR model, applying PCA resulted in mixed effects across evaluation metrics (Table S3). A modest yet insignificant improvement was observed in RMSE (*p*-value = 2.2 × 10^–1^) while both Spearman’s correlation coefficient and affine-transformed RMSE exhibited small drop (*p*-value = 6.0 × 10^–1^ and 1.6 × 10^–4^, respectively). Classification metrics also showed a mild decrease upon dimensionality reduction (*p*-value = 1.4 × 10^–4^ and 4.4 × 10^–4^ for *F*_1_-score and accuracy, respectively). The relatively minor performance differences observed demonstrate that EPIC is robust to dimensionality reduction and can exploit either the full feature space or a reduced representation of it. All in all, these results indicate that while PCA can offer modest benefits in specific regression metrics, it is not universally advantageous.

### 3.4 Feature Importance in the Regression Module

Since the Trigrams used in the regression module can be biologically interpretable as opposed to pLM-based embeddings, we performed SHAP analysis on the Trigrams + GBR model with optimized hyperparameters. SHAP values were computed for a representative cross-validation repeat and summarized using a beeswarm plot highlighting the top contributing sequence features (Figure 5).

**Figure 5:**
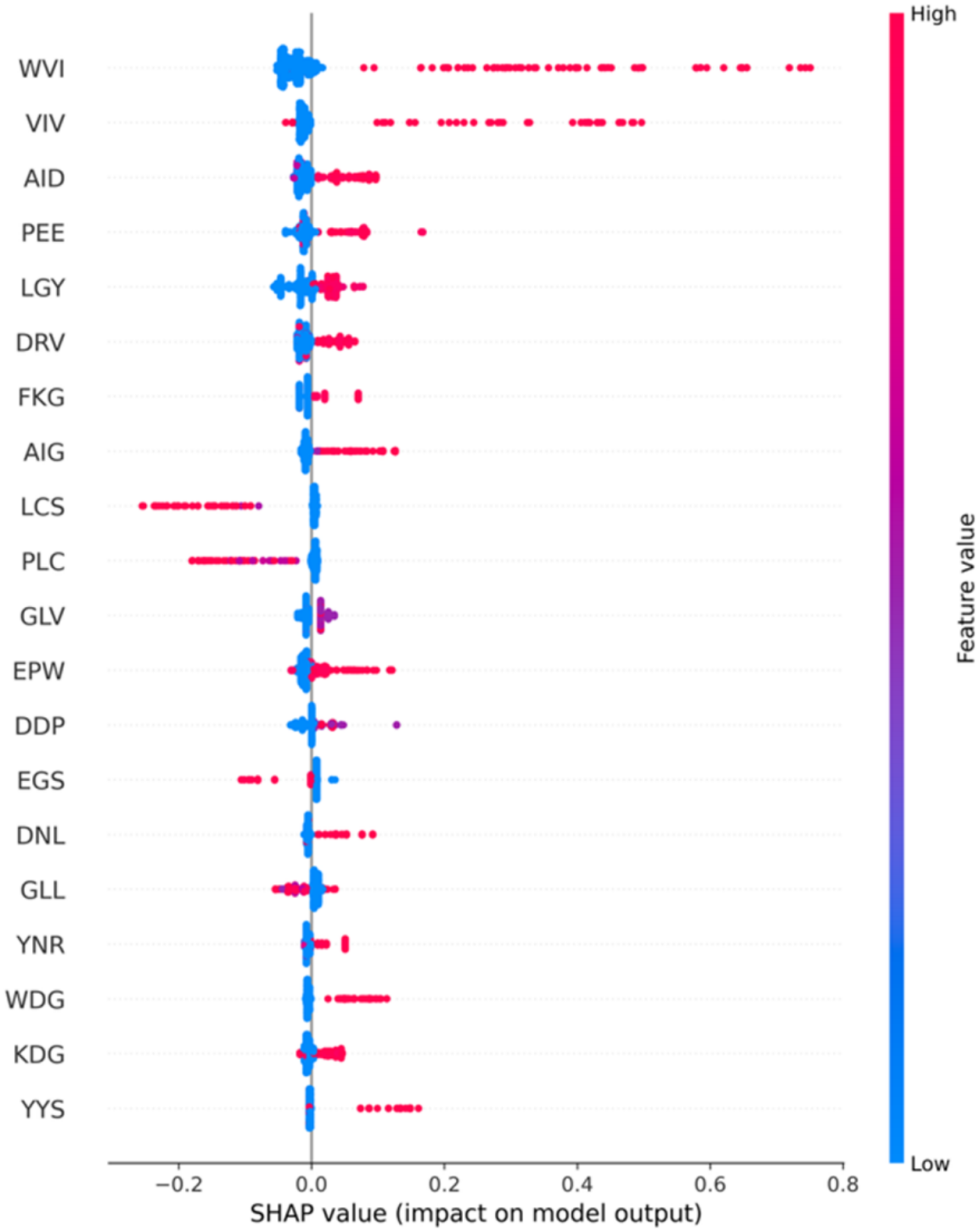
Beeswarm plot of SHAP values for the top 20 sequence features computed on a representative cross-validation repeat of the Trigrams + GBR regression model. Each point corresponds to one sample (relative activity value at a specific glucose concentration for a given enzyme) positioned by its impact on predicted relative activity. Point color indicates the feature value (low to high, shown in blue to red).

The most influential sequence features correspond to a small subset of specific amino-acid trigrams, including motifs such as WVI and VIV. Importantly, these trigrams exert directionally consistent effects, increasing predicted relative activity with their presence or higher frequency. The WVI trigram, when present, consistently appeared adjacent to the NEP motif where the glutamate corresponds to the conserved nucleophilic catalytic residue in GH1 BGLs.^54^ The proximity of this highly hydrophobic trigram to the active site assists in stabilizing substrate binding by acting as “gatekeeper residues” limiting the entrance of glucose to the substrate channel.^38,55^ Moreover, previous multiple sequence alignments studies suggested that the tryptophan residue is associated with increased glucose tolerance/stimulation.^28,38,56,57^ Notably, these associations were identified by our model, without explicitly encoding catalytic residues or prior mechanistic hypotheses, indicating that EPIC captures functionally relevant sequence context. As for VIV, this Trigram was observed in different sequence contexts: in a small subset of enzymes, it formed part of a “WVIV” motif located near the active site, whereas in others it occurred at distal positions with no consistent spatial relationship to catalytic residues. This lack of positional conservation suggests that, unlike WVI, the contribution of VIV is not readily attributable to a single structural or mechanistic role. Meanwhile, the narrow SHAP value distributions for most of the other trigrams indicate that their contributions are additive refinements rather than primary drivers, collectively modulating relative activity predictions. This behavior is consistent with the strong performance of Trigrams representations in the regression models and suggests that the sequence features determining the model’s predictive capacity for glucose response curves are distributed across the entire protein chain.

### 3.5 Evaluating EPIC’s Performance on Enzymes beyond the Original Dataset

To assess the robustness of EPIC beyond the original dataset, we evaluated both the classification and regression modules on a set of sequence-divergent and functionally distinct BGLs not used during model development. This external test set comprised: (i) a wild-type enzyme (Bgl2A) from a marine microbial metagenome,^58^ (ii) two single-point mutants of Bgl2A, (iii) its corresponding double mutant, and (iv) two ancestral sequences of the BGL from *Pyrococcus furiosus* (PfBGL) obtained via ancestral sequence reconstruction^26^ (Figure S6). The sequences span a wide range of sequence identity to the training data, with maximum pairwise identities ranging from 63.2% to 86.6% and average identity to the top 3 nearest neighbors between 62.5% and 68.6% (Tables S4 and S5).

We first evaluated EPIC’s classification module on the unseen enzymes to assess robustness of the discrete glucose-response class predictions (Table 3). The ProstT5 + SVM classifier correctly predicted the glucose-response class for Bgl2A and its single point mutants. This indicates strong generalization within a local sequence space around Bgl2A despite the lack of similar sequences in the original dataset. In contrast, the double mutant, experimentally assigned to Class I, was misclassified as Class III. Hence, the classification module faces difficulty capturing nonadditive mutational effects in a discrete label space. Among the PfBGL ancestral enzymes, one was correctly classified as Class II while the other was misclassified as Class II instead of Class III.

**Table 3:** Prediction Performance of EPIC with ProstT5 + SVM Classifier on Unseen Enzymes beyond the Original Dataset

unseen enzyme	true class	predicted class
Bgl2A	III	III
Bgl2A A22S	III	III
Bgl2A V224D	III	III
Bgl2A A22S/V224D	I	III
Ancestral Node 13	II	II
Ancestral Node 15	III	II

For regression, despite the substantial sequence divergence, EPIC, operated with Trigrams + GBR, successfully recapitulates the qualitative shape of glucose response curves across most unseen enzymes, including nonmonotonic behaviors. Particularly, Bgl2A and its single-point mutants exhibit reasonable agreement between predicted and observed curves, with high Spearman rank correlations (ρ = 0.72–0.92) and fair affine-transformed RMSE values (Figure 6A–C). This indicates that the model generalizes well to significant sequence perturbations around an unseen scaffold, preserving both trend direction and relative response shape. The double mutant Bgl2A A22S/V224D represents a more challenging case. While the regression model captures the inhibitory trend at high glucose concentrations, it displays stimulation at lower concentrations that was not observed experimentally (Figure 6D). The predicted trend is comparable to those followed by Bgl2A and its single point mutants indicating that the regression model, similar to the classification model, may not have learned the epistatic effects associated with the mutations. This is to be expected given the size of the training data, and the scarcity of training samples with multiple mutations (<20% of the data).

**Figure 6:**
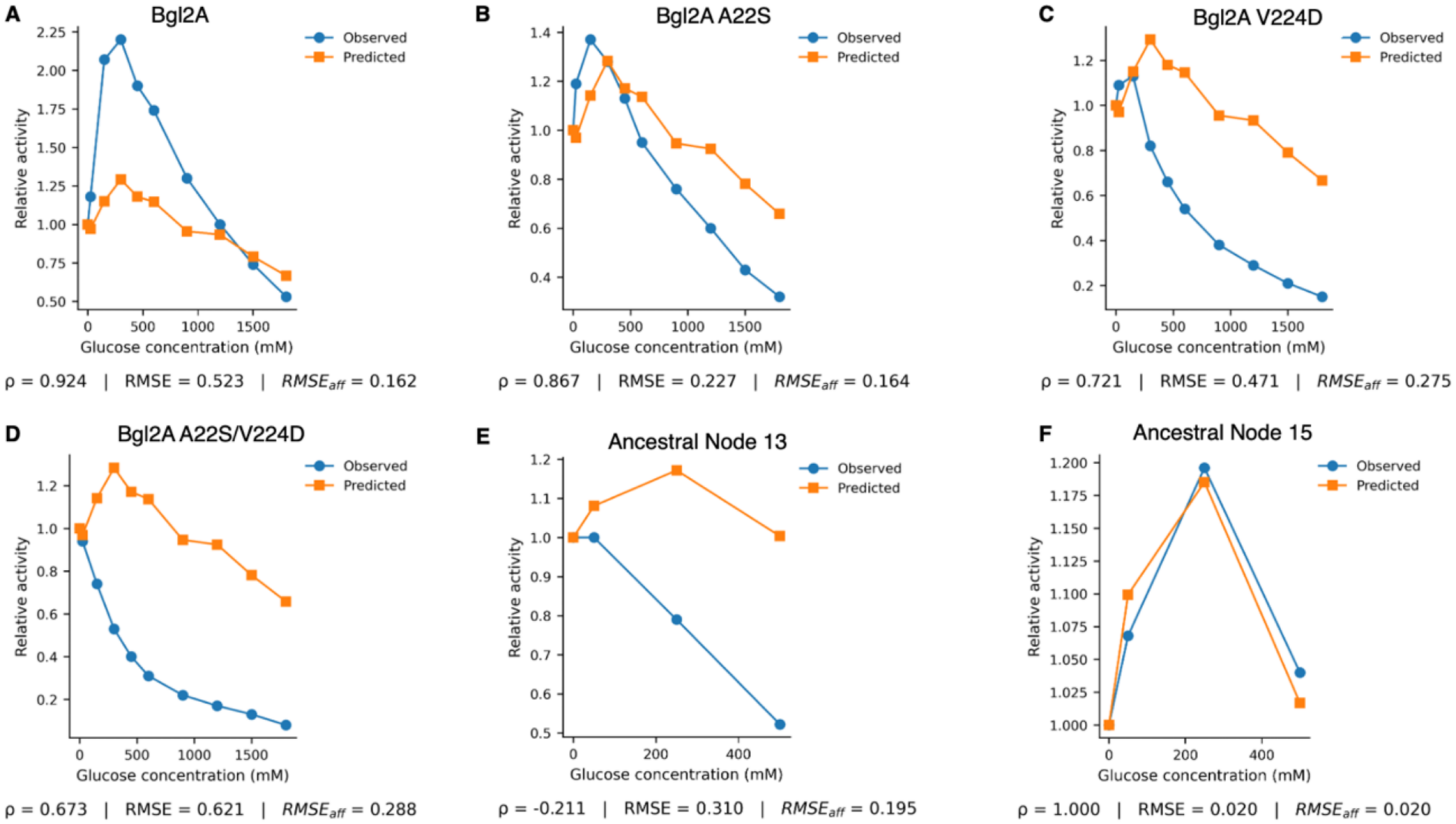
Prediction performance of EPIC with Trigrams + GBR on unseen enzymes beyond the original dataset. Panels include (A) the wild-type Bgl2A enzyme, (B–C) single point mutants of Bgl2A, (D) a double mutant of Bgl2A, and (E, F) reconstructed PfBGL ancestral sequences. For each enzyme, Spearman’s rank correlation coefficient, RMSE, and affine-transformed RMSE are reported below the plot. Observed data refers to experimentally determined values either taken directly from published papers^58^ (A–D) or measured in this study (E, F).

Notably, the two ancestral enzymes, with higher sequence identity to the training set show mixed behavior: one ancestral sequence exhibits an incorrect glucose-response behavior, while the other is predicted with near-perfect agreement (Figure 6E,F). These results underscore the challenge in predicting the behavior of ancestral sequences, which may exhibit transitional functional behaviors not well represented in contemporary training data.

Overall, these results demonstrate that EPIC generalizes well to most of the unseen enzymes, especially when evaluated using Spearman’s coefficient and affine-transformed RMSE in the regression module that emphasize curve shape over absolute scaling. In both modules, performance degrades, though marginally, with increasing mutational complexity, highlighting both the strengths and current limits of EPIC. Interestingly, the two ancestral enzymes exhibit opposite behavior across the regression and classification tasks: one ancestral sequence is predicted with high fidelity by the regression model but misclassified, whereas the other shows the opposite trend. This asymmetry highlights the complementary roles of classification and regression within EPIC: regression enables the recovery of functional trends while classification provides a coarse discretization that may differ near class boundaries.

### 3.6 Limitations and Future Directions

Several limitations of the current EPIC framework should be considered when interpreting its performance. First, at 105 unique BGL sequences, the dataset remains relatively small and imbalanced, especially for the classification task. In addition, because the dataset includes both wild type enzymes and mutants, highly similar sequences may appear in different cross-validation folds. This may lead to optimistic performance estimates, particularly when test enzymes differ from training enzymes by only one or a few mutations. To examine this effect, we evaluated predictions separately for wild-type and mutant enzymes within the original repeated cross-validation framework (Table S6). Both classification and regression performance were higher for mutants than for wild-type enzymes confirming that closely related variants are easier to predict. However, the model retained predictive signal on wild-type enzymes, indicating that performance was not exclusively driven by mutant entries. Although the current cross-validation scheme is an endeavor to circumvent this effect, a more rigorous sequence-similarity-based split would be needed as more data become available.^59^ Finally, EPIC does not fully account for experimental variability across studies. Despite of the inclusion of substrate concentration, other assay variables such as pH and temperature were not considered because these parameters are often not reported in the literature, and data for a given sequence across multiple experimental assay conditions are rarely available. Future efforts to generate enzyme–product datasets under standardized assay conditions and across a range of experimental conditions will help improve model performance and generalizability.

Second, the model could benefit from incorporating structural features pertaining to active site dynamics. Unfortunately, experimentally resolved structures are unavailable for a substantial fraction of sequences included in this study. Moreover, prior work has shown that predicted structures generated using tools such as ESMFold do not consistently improve performance in enzyme activity parameter prediction relative to sequence-derived representations alone, presumably due to error propagation and the difficulty of modeling the complex conformational dynamics involved in enzyme reactions.^7,60^ For example, the CatPred-*K*_*i*_ model tasked with the prediction of the inhibition constant for enzymes showed a mere 0.002 improvement in test set *R* ^2^ upon incorporating features from ESMFold-predicted structures encoded using an equivariant graph neural network. Rather than using entire 3D-structures, a specific description of enzyme–substrate binding pockets and active-site amino acids could potentially be more informative.^61^ As more accurate and comprehensive enzyme structure and dynamics information becomes available, our model can be extended to incorporate these features and improve prediction accuracy.

One practical improvement would be to automate the data extraction from the relative activity curves against product concentrations. Since these relationships are often only reported as plots, they require manual digitization (as performed in this study) which becomes a bottleneck for regression-based modeling. Image-mining tools could be used to extract data points directly from published graphs, offering larger training sets for regression. Lastly, the framework could be extended to other enzyme–product pairs, further expanding EPIC’s utility for evaluating product effects on sequences of interest in enzyme mining and engineering.

## 4 Conclusion

This work introduces and evaluates EPIC, an ML framework used to predict the glucose response classes and profiles of BGL enzymes primarily from amino acid sequences and substrate concentration. Compared to a sequence-identity-based baseline, EPIC provides a superior output over extensive cross-validation while simultaneously preserving response trends. When tested on enzymes beyond the original dataset, the model generalized to most unseen sequences and a wide variety of glucose responses. In combination, these results establish EPIC as a scalable framework for modeling relative BGL activity under enzyme–product interactions while simultaneously providing both classification and regression predictions.

## Supplementary Material





## Data Availability

The datasets, scripts, and source code for EPIC are made publicly available at: https://github.com/ali-malli/EPIC.

## Notes

Assay-dependent glucose behavior (Figure S1), classification results (Figures S2–S4), regression results (Figure S5), principal component analysis (Table S3), phylogenetic tree (Figure S6), external test set (Tables S4–S5), and wild type and mutant performance (Table S6) (PDF)
EPIC’s classification dataset (Table S1); EPIC’s regression dataset (Table S2) (XLSX)
