# Supplementary material for: EPIC: A Machine-Learning Framework for Product-Dependent Behavior in β‑Glucosidases
Source: ACS Omega. 2026 Jul 2;11(28):42759–71. doi: 10.1021/acsomega.6c04226 (PMC13393188; doi:10.1021/acsomega.6c04226)
Supplement: Supplementary file 1 [file ao6c04226_si_001.pdf]

Supporting Information

EPIC: A machine-learning framework for product-dependent behavior in β-glucosidases

Ali Malli[1], Denys Vasyutyn[1], Anuj Majumder[1], Jin Ryoun Kim[1,*]
[1] Department of Chemical and Biomolecular Engineering, New York University, 6 MetroTech Center, Brooklyn, NY 11201, United States

*** Corresponding Author:** jin.kim@nyu.edu

Supporting Information – List of Contents

Figure S1 - Representative BGLs (an alkaline BGL identified from metagenomic libraries, AS-Esc10[1], and an ancestral BGL selected from a phylogenetic tree near *Pyrococcus furiosus* BGL, Node 15[2]) whose response class changes across substrate concentrations, illustrating the context dependence of glucose-response behavior. The graph for AS-Esc101 was constructed based on data from the paper[1], whereas the graph for Node 15 was generated using our experimental data obtained in this study.

Figure S2 – Representative confusion matrices for the (A) sequence-identity baseline and (B) ProstT5 + SVM classifier

Figure S3 – Distributions of (A) macro-average F_1-score and (B) overall accuracy across 20 repeats of 5-fold cross-validation for the three-class classification task. Panels A and B compare the ProstT5-based RF classifier against the sequence-identity baseline. p-values are from a paired two-sided Wilcoxon signed-rank test across repeats.

Figure S4 – Multidimensional spacing depicting the sequence-identity space

Figure S5 – Representative outputs for the (A, B, C) Trigrams + GBR and (D, E, F) ProstT5 + ExtraTrees regressors. Reported activity refers to values taken directly from published papers. Model predictions are shown for representative enzymes exhibiting good (left), intermediate (middle), and poor (right) predictive performance under the regression models.

Figure S6 – The phylogenetic tree used to reconstruct the two ancestral BGL sequences on Nodes 13 and 15

Table S1 – EPIC's classification dataset (.xlsx file)

Table S2 – EPIC's regression dataset (.xlsx file)

Table S3 – Effect of PCA on EPIC's performance. The metrics represent mean performance across 20 repeats of 5-fold cross-validation. The change in RMSE and RMSEaff are reported with inverted signs so that the positive values consistently correspond to improved performance

Table S4 – Amino acid sequences of the unseen enzymes relative to the training set

Table S5 – Sequence identity of unseen enzymes relative to the training set

Table S6 – Performance metrics for the optimal classification (ProstT5 + SVM) and regression (Trigrams + GBR) models broken down by wild type and mutant enzymes. The metrics represent mean performance and the standard deviation across 20 repeats of 5-fold cross-validation.

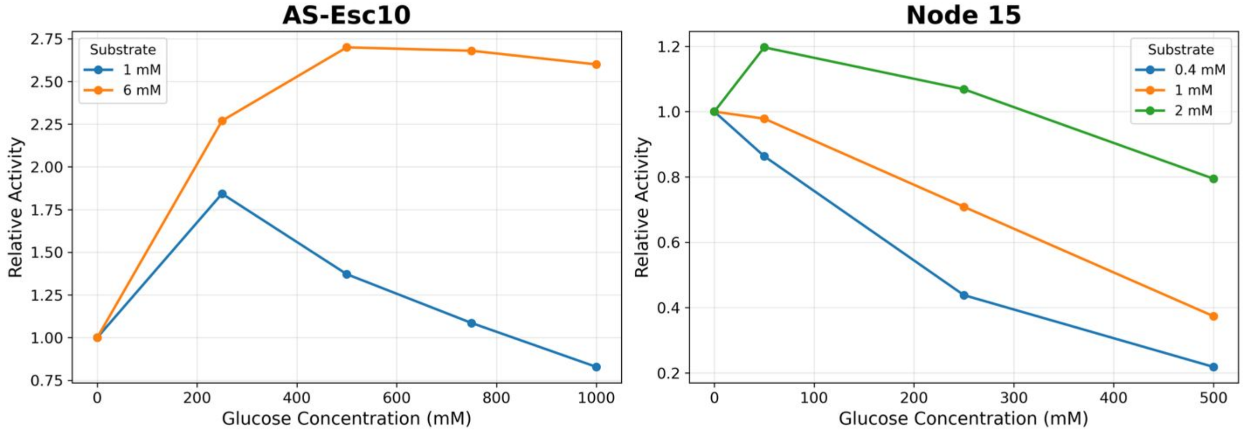

Figure S1 - Representative BGLs (an alkaline BGL identified from metagenomic libraries, AS-Esc10[1], and an ancestral BGL selected from a phylogenetic tree near *Pyrococcus furiosus* BGL, Node 15[2]) whose response class changes across substrate concentrations, illustrating the context dependence of glucose-response behavior. The graph for AS-Esc101 was constructed based on data from the paper[1], whereas the graph for Node 15 was generated using our experimental data obtained in this study.

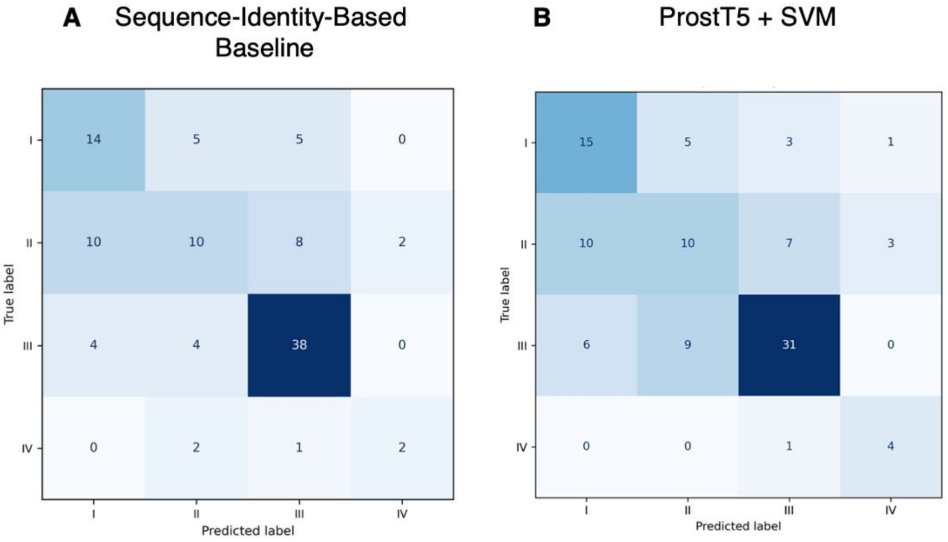

Figure S2 – Representative confusion matrices for the (A) sequence-identity baseline and (B) ProstT5 + SVM classifier

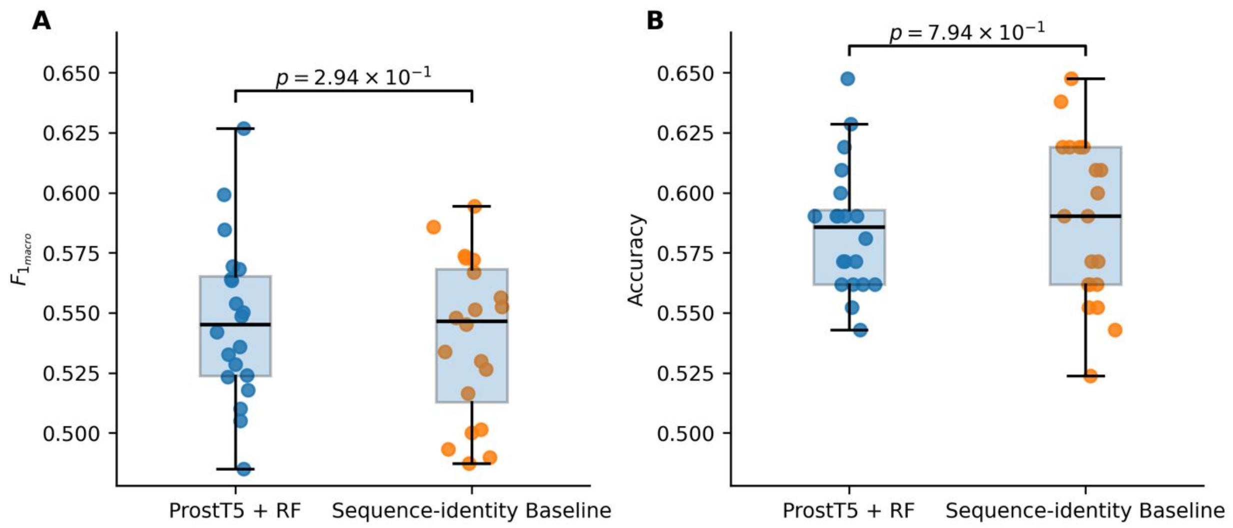

Figure S3 – Distributions of (A) macro-average F_1-score and (B) overall accuracy across 20 repeats of 5-fold cross-validation for the three-class classification task. Panels A and B compare the ProstT5-based RF classifier against the sequence-identity baseline. *p*-values are from a paired two-sided Wilcoxon signed-rank test across repeats.

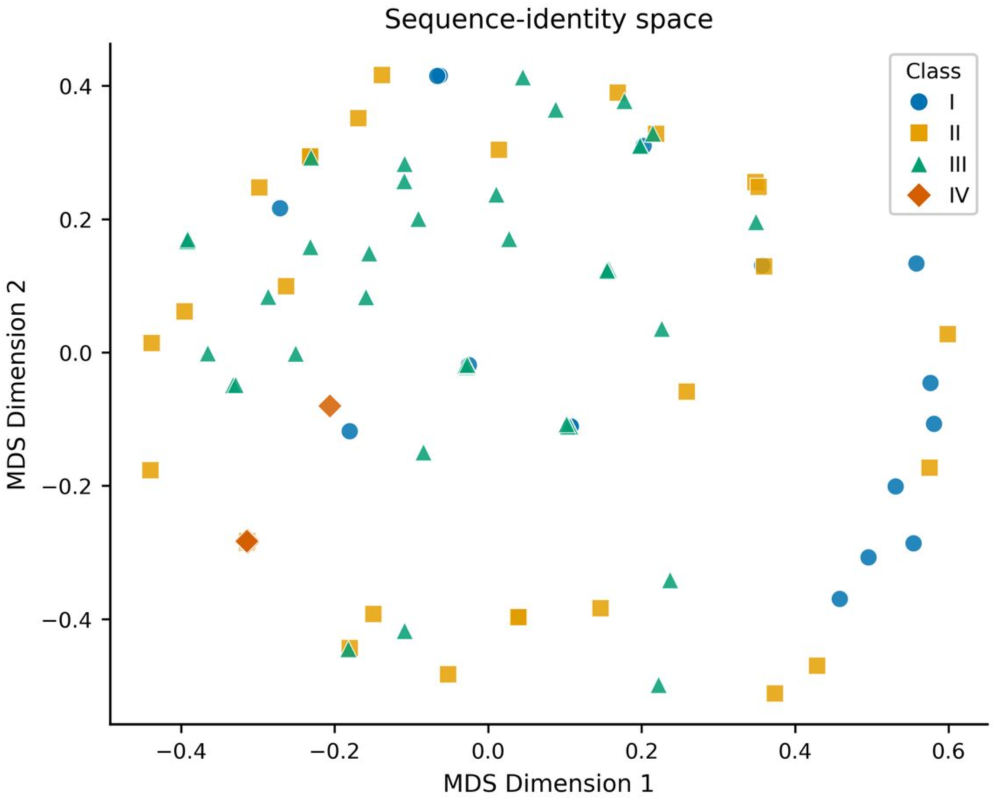

Figure S4 – Multidimensional spacing depicting the sequence-identity space

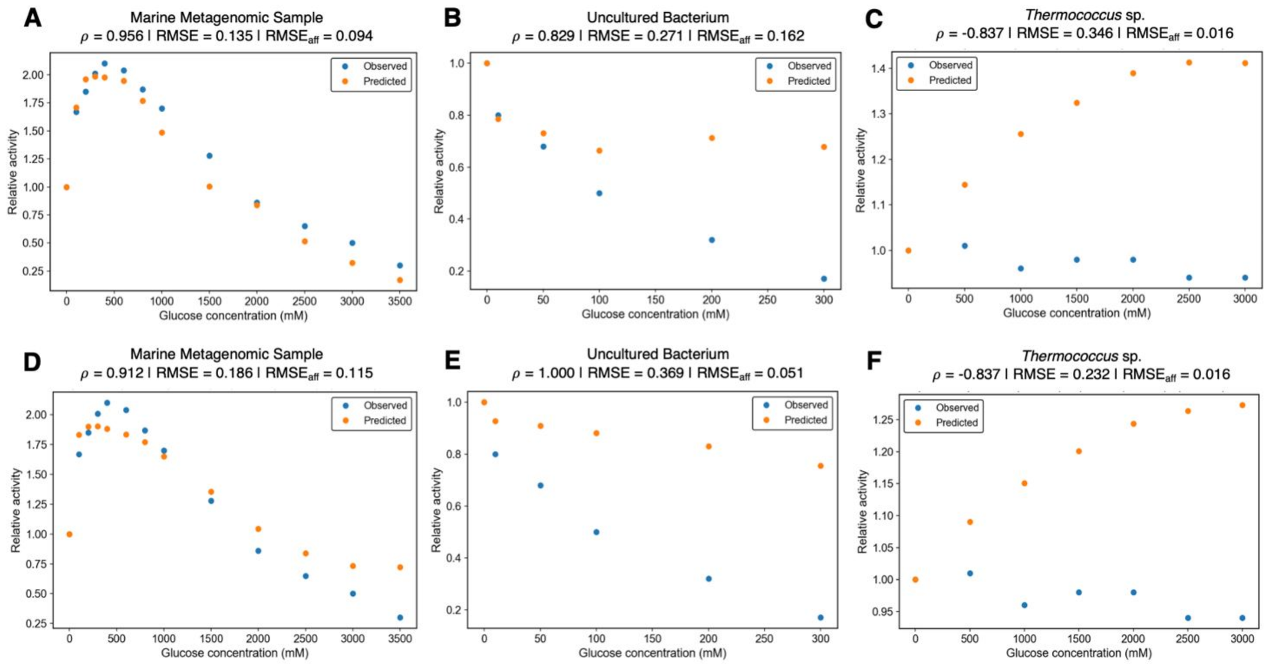

Figure S5 – Representative outputs for the (A, B, C) Trigrams + GBR and (D, E, F) ProstT5 + ExtraTrees regressors. Observed activity refers to values taken directly from published papers[3–5]. Model predictions are shown for representative enzymes exhibiting good (left), intermediate (middle), and poor (right) predictive performance under the regression models.

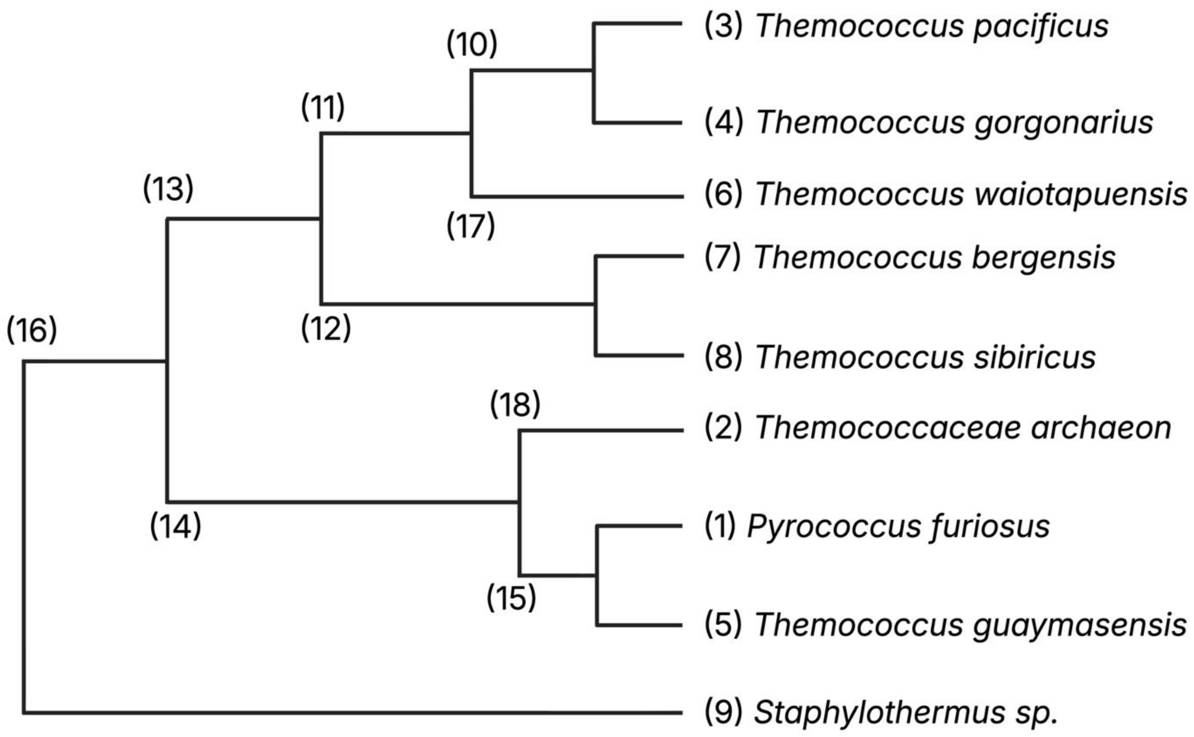

Figure S6 – The phylogenetic tree used to reconstruct the two ancestral BGL sequences on Nodes 13 and 15

Table S3 – Effect of PCA on EPIC's performance. The metrics represent mean performance across 20 repeats of 5-fold cross-validation.

Task	Model	Metrics	Metrics		Change in performance
			Before PCA	After PCA	
Classification	ProstT5 + SVM	F_1-score	0.577	0.555	− 0.022
		Accuracy	0.593	0.569	− 0.024
Regression	Trigrams + GBR	ρ	0.662	0.658	− 0.004
		RMSE	0.402	0.396	− 0.006
		$RMSE_{aff}$	0.149	0.158	+ 0.009

Table S4 – Amino acid sequences of the unseen enzymes relative to the training set

Unseen enzyme	Amino Acid Sequence
Bgl2A	MTKISLPTCSPLLTKEFIYGVATSSFQIEGGSAHRLPCIWDTFC<br>DTPGKIADNSNGHVACDHYNNWKQDIDLIESLGVDAYRLSIS<br>WPRVITKSGELNPEGVKFYTDILDELKKRNIKAFVTLYHWDL<br>PQHLEDEGGWLNRETAYAFAHYVDLITLAFGDRVHSYATLN<br>EPFCSAFLGYEIGIHAPGKVGKQYGRKAAHHLLLAHGLAMTV<br>LKQNSPTTLNGIVLNFTPCYSISEDADDIAATAFADDYLNQWY<br>MKPIMDGTYPAIIEQLPSAHLPDIHDGDMAIISQSIDYLGINYY<br>TRQFYKAHPTEIYEPIEPTGPLTDMGWEIYPKSFTELLVTLNNT<br>YTLPPIFITENGAAMPDSYNNGEINDVDRLDYYNSHLNAVHN<br>ATEQGVRIDGYFAWSLMDNFEWAEGYLKRFGIVYVDYSTQQ<br>RTIKNSGLAYKALISNR
Bgl2A A22S	MTKISLPTCSPLLTKEFIYGVSTSSFQIEGGSAHRLPCIWDTFCD<br>TPGKIADNSNGHVACDHYNNWKQDIDLIESLGVDAYRLSISW<br>PRVITKSGELNPEGVKFYTDILDELKKRNIKAFVTLYHWDLPQ<br>HLEDEGGWLNRETAYAFAHYVDLITLAFGDRVHSYATLNEPF<br>CSAFLGYEIGIHAPGKVGKQYGRKAAHHLLLAHGLAMTVLK<br>QNSPTTLNGIVLNFTPCYSISEDADDIAATAFADDYLNQWYM<br>KPIMDGTYPAIIEQLPSAHLPDIHDGDMAIISQSIDYLGINYYTR<br>QFYKAHPTEIYEPIEPTGPLTDMGWEIYPKSFTELLVTLNNTYT<br>LPPIFITENGAAMPDSYNNGEINDVDRLDYYNSHLNAVHNAT<br>EQGVRIDGYFAWSLMDNFEWAEGYLKRFGIVYVDYSTQQRT<br>IKNSGLAYKALISNR
Bgl2A V224D	MTKISLPTCSPLLTKEFIYGVATSSFQIEGGSAHRLPCIWDTFC<br>DTPGKIADNSNGHVACDHYNNWKQDIDLIESLGVDAYRLSIS

	WPRVITKSGELNPEGVKFYTDILDELKKRNIKAFVTLYHWDL<br>PQHLEDEGGWLNRETAYAFAHYVDLITLAFGDRVHSYATLN<br>EPFCSAFLGYEIGIHAPGKVGKQYGRKAAHHLLLAHGLAMTV<br>LKQNSPTTLNGIDLNFTPCYSISEDADDIAATAFADDYLNQWY<br>MKPIMDGTYPAIIEQLPSAHLPDIHDGDMAIISQSIDYLGINYY<br>TRQFYKAHPTEIYEPIEPTGPLTDMGWEIYPKSFTELLVTLNNT<br>YTLPPIFITENGAAMPDSYNNGEINDVDRLDYYNSHLNAVHN<br>ATEQGVRIDGYFAWSLMDNFEWAEGYLKRFGIVYVDYSTQQ<br>RTIKNSGLAYKALISNR
Bgl2A A22S/V224D	MTKISLPTCSPLLTKEFIYGVSTSSFQIEGGSAHRLPCIWDTFCD<br>TPGKIADNSNGHVACDHYNNWKQDIDLIESLGVDAYRLSISW<br>PRVITKSGELNPEGVKFYTDILDELKKRNIKAFVTLYHWDLPQ<br>HLEDEGGWLNRETAYAFAHYVDLITLAFGDRVHSYATLNEPF<br>CSAFLGYEIGIHAPGKVGKQYGRKAAHHLLLAHGLAMTVLK<br>QNSPTTLNGIDLNFTPCYSISEDADDIAATAFADDYLNQWYM<br>KPIMDGTYPAIIEQLPSAHLPDIHDGDMAIISQSIDYLGINYYTR<br>QFYKAHPTEIYEPIEPTGPLTDMGWEIYPKSFTELLVTLNNTYT<br>LPPIFITENGAAMPDSYNNGEINDVDRLDYYNSHLNAVHNAT<br>EQGVRIDGYFAWSLMDNFEWAEGYLKRFGIVYVDYSTQQRT<br>IKNSGLAYKALISNR
Ancestral Node 13	MKFPKDFLFGYSWSGFQFEMGLPGSEVPNSDWWVWVHDKE<br>NIASGLVSGDLPENGPAYWHLYKQDHDIAESLGMDAIRGGIE<br>WARIFPKPTFDVKVDVEKDEDGNIVSVDVPESAIEELEKIADM<br>EALEHYRKIYSDWKERGKTFILNLYHWPLPLWLHDPIKVRRL<br>GPDRAPSGWLDDRSVVEFAKFAAFVAYHLDDLVDMWSTMN<br>EPNVVYEQGYTRPKSGFPPGYLSFEASEKAKMNLIQAHARAY<br>DAIKEYSDKPVGIIYAYTWLDPLNEEIEDEVREIRKRDLYSFVD<br>AVHSGKSASAEEREDLKGRVDWIGVNYYSRLAFGKVNGHIV<br>PLPGYGFSSERGGYAKSGRPASDFGWEIYPEGLEKLLRELSKR<br>YNLPMMITENGIADAADRYRPYYLVSHLKAVYNAMKEGAD<br>VRGYLHWSLTDNYEWAQGFRMRFGLVYVDFETKKRYLRPS<br>ALVFREIATRKEIPEELEHLADLNALTRR
Ancestral Node 15	MKFPKDFLFGYSWSGFQFEMGLPGSEVPNSDWWVWVHDKE<br>NIASGLVSGDLPENGPAYWHLYKQDHDIAESLGMDAIRGGIE<br>WARIFPKPTFDVKVDVEKDEDGNIVSVDVPESAIEELEKLAD<br>MEALEHYRKIYSDWKERGKTFILNLYHWPLPLWLHDPIAVRR<br>LGPDRAPSGWLDERSVVEFVKFAAFVAYHLDDLVDMWSTM<br>NEPNVVYEQGYTRPNSGFPPGYLSFEASEKAKMNLIQAHARA<br>YDAIKEYSEKPVGIIYAFAWHDPLNEEVEDEVKEIRKRDYEFV<br>TAVHSGKSATAGEREDLKGKLDWIGVNYYSRLAYGKKNGHI<br>VPLPGYGFMSERGGFAKSGRPASDFGWEIYPEGLENLLRELN<br>NRYELPMMITENGIADAADRYRPYYLVSHLKAVYNAMKEGA<br>DVRGYLHWSLTDNYEWAQGFRMRFGLVYVDFETKKRYLRP<br>SALVFREIATRKEIPEELAHLADLNALTRR

Table S5 – Sequence identity of unseen enzymes relative to the training set

Unseen enzyme	Maximum Sequence Identity	Average Sequence Identity with k = 3 Neighbors
Bgl2A	63.7%	63.0%
Bgl2A A22S	63.5%	62.7%
Bgl2A V224D	63.5%	62.7%
Bgl2A A22S/V224D	63.2%	62.5%
Ancestral Node 13	83.3%	67.3%
Ancestral Node 15	86.6%	68.6%

Table S6 – Performance metrics for the optimal classification (ProstT5 + SVM) and regression (Trigrams + GBR) models broken down by wild type and mutant enzymes. The metrics represent mean performance and the standard deviation across 20 repeats of 5-fold cross-validation.

Enzyme	Classification Metrics		Regression Metrics		
	F_1-score	Accuracy	ρ	RMSE	$RMSE_{aff}$
Wild Type	0.401 ± 0.027	0.548 ± 0.031	0.639 ± 0.021	0.414 ± 0.013	0.163 ± 0.003
Mutant	0.602 ± 0.042	0.566 ± 0.043	0.774 ± 0.016	0.335 ± 0.005	0.129 ± 0.004

References

1. Biver, S., Stroobants, A., Portetelle, D. & Vandenbol, M. Two promising alkaline β-glucosidases isolated by functional metagenomics from agricultural soil, including one showing high tolerance towards harsh detergents, oxidants and glucose. *J. Ind. Microbiol. Biotechnol.* **41**, 479–488 (2014).
2. Erkanli, M. E. *et al.* A Three-Module Machine Learning Framework for Protein Sequence- and Temperature-Dependent kcat/Km Prediction in β-Glucosidases. *ACS Synth. Biol.* https://doi.org/10.1021/acssynbio.5c00257 (2025) doi:10.1021/acssynbio.5c00257.
3. Cao, L. *et al.* Engineering a novel glucose-tolerant β-glucosidase as supplementation to enhance the hydrolysis of sugarcane bagasse at high glucose concentration. *Biotechnol. Biofuels* **8**, 202 (2015).
4. Cao, L. *et al.* Enhancing the Thermostability of Highly Active and Glucose-Tolerant β-Glucosidase Ks5A7 by Directed Evolution for Good Performance of Three Properties. *J. Agric. Food Chem.* **66**, 13228–13235 (2018).
5. Sinha, S. K., Prakash Reddy, K. & Datta, S. Understanding the glucose tolerance of an archaeon β-glucosidase from *Thermococcus* sp. *Carbohydr. Res.* **486**, 107835 (2019).
